# High brain acid soluble protein 1(BASP1) is a poor prognostic factor for cervical cancer and promotes tumor growth

**DOI:** 10.1186/s12935-017-0452-4

**Published:** 2017-10-24

**Authors:** Huiru Tang, Yan Wang, Bing Zhang, Shiqiu Xiong, Liangshuai Liu, Wei Chen, Guosheng Tan, Heping Li

**Affiliations:** 1 Department of Gynecology & Obstetrics, Peking University Shenzhen Hospital, Shenzhen, 518036 People’s Republic of China; 2 Shenzhen Key Laboratory of Gynecological Diagnostic Technology Research, Shenzhen, 518036 People’s Republic of China; 3 Department of Gynecology and Obstetrics, Nanfang Hospital, Southern Medical University, Guangzhou, 510515 People’s Republic of China; 4 Department of Nuclear Medicine, The First Affiliated Hospital of Sun Yat-sen University, Guangzhou, 510080 People’s Republic of China; 5 Department of Biochemistry, University of Leicester, Leicester, LE1 7RH UK; 6 Department of Interventional Radiology, The First Affiliated Hospital of Sun Yat-sen University, Guangzhou, 510080 People’s Republic of China; 7 Department of Medical Oncology, The First Affiliated Hospital of Sun Yat-sen University, 58 Zhongshan Road II, Yuexiu District, Guangzhou, 510080 People’s Republic of China

**Keywords:** BASP1, Cervical cancer, Prognosis, Tumor growth

## Abstract

**Background:**

The aim of this study was to determine whether brain abundant membrane attached signal protein 1 (BASP1) is a valuable prognostic biomarker for cervical cancer and whether BASP1 regulates the progression of cervical cancer.

**Methods:**

Quantitative real-time PCR, western blotting, and immunohistochemistry were used to determined BASP1 levels. Statistical analyses were used to examine whether BASP1 was a prognostic factor for patients with cervical cancer. The MTT assay, colony formation assay, cell cycle assay, anchorage-independent growth assay, and a tumor xenograft model were used to determine the role of BASP1 in the proliferation and tumorigenicity of cervical cancer.

**Results:**

Brain abundant membrane attached signal protein 1 was upregulated in cervical cancer tissues and cells, and BASP1 expression levels were higher in patients that had died during follow-up compared with those that survived. There was a positive correlation between BASP1 expression and clinical stage (p < 0.001), T classification (p < 0.001), N classification (p < 0.05), and survival or mortality (p < 0.05). Patients with higher BASP1 expression had a shorter overall survival time. Cox regression analysis shown BSAP1 was an unfavorable prognostic factor for patients with cervical cancer. Overexpression of BASP1 promoted the proliferation of cervical cancer and its colony formation ability, accelerated cell cycle progression, and enhanced tumorgenicity. *BASP1* knockdown inhibited the proliferation of cervical cancer and its colony formation ability, suppressed cell cycle progression, and decreased tumorgenicity.

**Conclusions:**

The results showed that BASP1 not only is a novel prognostic factor for patients with cervical cancer, but also promotes the proliferation and tumorigenicity of cervical cancer.

**Electronic supplementary material:**

The online version of this article (doi:10.1186/s12935-017-0452-4) contains supplementary material, which is available to authorized users.

## Background

In recent decades, certain prognostic factors and therapeutic targets for cervical cancer have been found. For example, weak pp125FAK expression correlates with pelvic lymph node metastasis and recurrent disease, and is a favorable factor for patients with cervical cancer: the overall survival of patients with high and moderate pp125FAK levels was longer than those with weak PP125FAK expression [1]. Msi1 is upregulated in patients with cervical cancer, and promotes the proliferation of cervical cancer by directly inhibiting p21, p27, and p53 [2]. However, cervical cancer remains the fourth most common cancer in women worldwide. Its morbidity has decreased in some countries, probably because of progress in early diagnosis and prevention. In China, the morbidity of cervical cancer is 8.98/100,000 and the mortality is 2.13/100,000, according to data from the 2012 Chinese Cancer Registry Annual Report [3]. This suggested that new prognostic factors and therapeutic approaches should be developed.

We used publically available gene expression profiles of cervical cancer tissues and normal cervical tissues (GSE9750) to screen for genes that regulate the progression of cervical cancer, and found that *BASP1* (encoding brain abundant membrane attached signal protein 1) was upregulated in cervical cancer tissues. BASP1, which is also known as NAP-22 or CAP-23 [4], can interact with Wilms tumor 1 (WT1). WT1 is a Wilms’ tumor suppressor protein that plays an important role in nephrogenesis and hematopoiesis [5]. BASP1 serves as a transcriptional co-suppressor to inhibit transcriptional activity of WT1, suggesting BASP1 regulates the function of WT1 in development [6, 7]. Further analysis revealed that the N-terminus of BASP1 could be myristoylated; myristoylated BASP1 interacted with oleate-activated transcription factor PIP2, which recruits histone deacetylase histone deacetylase 1 (HDAC1) to the promoter regions of WT1-dependent target genes, causing transcriptional repression [8]. Toska and colleagues observed that BASP1 also interacted with Prohibitin to recruit BRG1 to the promoter regions of WT1-dependent target genes, causing coactivator p300/CBP to dissociate from the promoter regions to inhibit target gene expression; the interaction between BASP1 and Prohibitin is also critical for the recruitment of PIP2 and HDAC1 to the target genes of WT1 [9]. These findings suggested BASP1 plays an important role in development. However, the role of BASP1 in cervical cancer has not been reported.

In this study, we analyzed the relationship between BASP1 expression and clinicopathological parameters in patients with cervical cancer, and studied the role of BASP1 in the cervical cancer growth. We found that BASP1 is a new prognostic factor for cervical cancer, and promotes tumor growth.

## Results

### BASP1 is upregulated in cervical cancer tissues

**Fig. 1:**
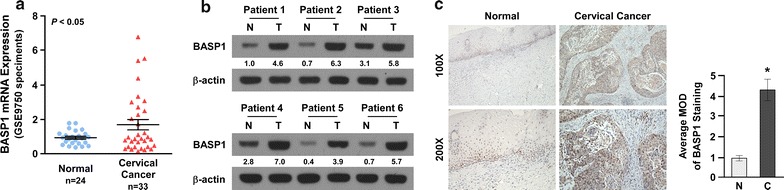
BASP1 was upregulated in cervical cancer tissues. **a** BASP1 expression was upregulated in cervical cancer tissues compared with normal cervical epithelium tissues; p < 0.05. **b** Western blot showing BASP1 levels in cervical cancer tissues and adjacent normal cervical epithelium tissues. **c** Immunohistochemistry assay showing BASP1 levels in cervical cancer tissues and normal cervical epithelium tissues. BASP1 staining of normal cervical epithelium tissue (left), BASP1 staining of cervical cancer tissue (right) (×100 and ×200, respectively)

To investigate whether BASP1 regulates the development and progression of cervical cancer, we explored BASP1 expression in cervical cancer tissues and normal cervical epithelium tissues. Analysis of gene expression profiles (GSE9750, Fig. 1a) showed that *BASP1* was significantly upregulated in cervical tissues compared with normal cervical tissue. Six paired cervical cancer tissues and matched adjacent normal cervical tissues were used to examine BASP1 protein levels. Western blotting showed that BASP1 levels were higher in cervical cancer tissues than in matched adjacent normal cervical tissues (Fig. 1b). Immunohistochemical analysis also revealed that BASP1 was upregulated in cervical cancer tissues (Fig. 1c). These results suggested that BASP1 is upregulated in cervical cancer tissues.

### BASP1 levels correlate with clinical aggressiveness of cervical cancer

**Fig. 2:**
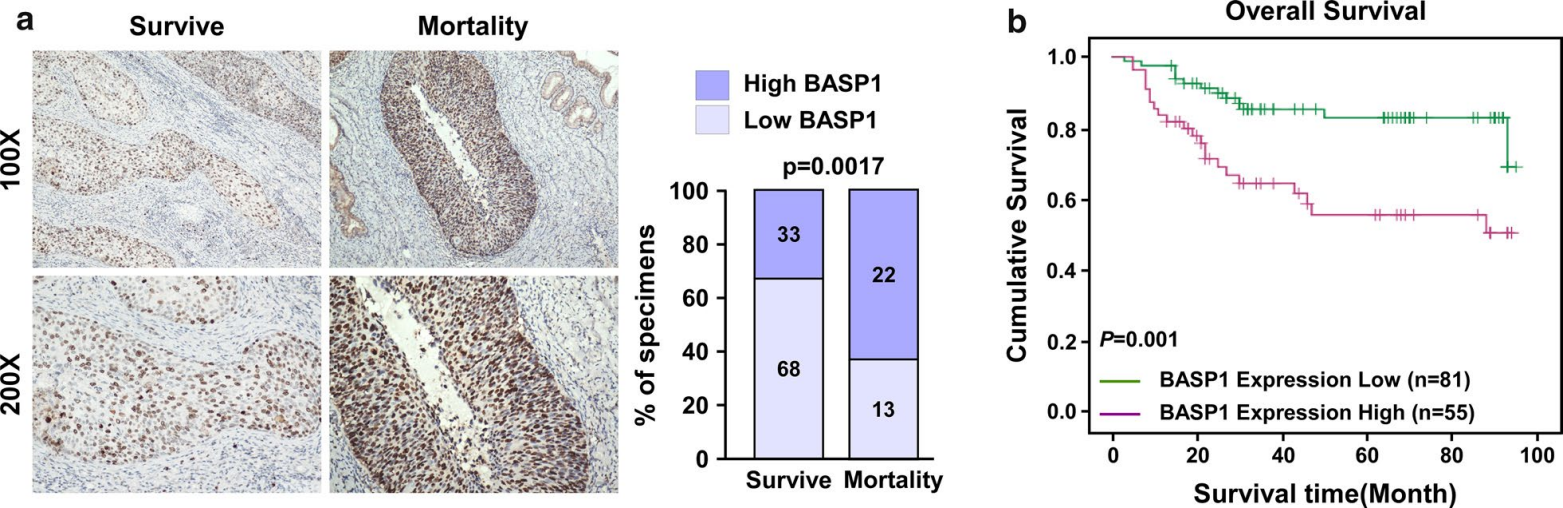
Patients with higher BASP1 expression had shorter overall survival time. **a** Immunohistochemistry assay showing BASP1 levels in tissues of patients who have died in the follow-up time and the tissues of patients who survived during the follow-up time. BASP1 staining of cervical cancer tissue from survival patients (left), BASP1 staining of cervical cancer tissue from died patients (right) (×100 and ×200, respectively). Statistical analysis of the number of patients with high BASP1 expression and low BASP1 expression in patients who have died and patients who survived, respectively; p = 0.0017. **b** Kaplan–Meier survival curve and log-rank test for patients with cervical cancer classified as showing either high or low BASP1 expression; p = 0.001

To determine the relationship between BASP1 levels and clinicopathological parameters, and whether BASP1 could serve as a new independent prognostic factor, we determined BASP1 levels in a cohort of 136 paraffin-embedded archived cervical cancer tissues using immunohistochemistry. Positive staining for BASP1 was observed in 98.5% (134/136) of samples, with low BASP1 staining in 59.6% (81/136) samples and high BASP1 staining in 40.4% (55/136) of the samples (Additional file 1: Table S2). BASP1 levels were higher in patients who had died during follow-up than in those had survived during follow-up (Fig. 2a). BASP1 levels were low in 67.3% (68/101) of surviving patients, and high in 32.7% (33/101). However, BASP1 levels were low in 37.1% (13/35) of the patients who died and high in 62.9% (22/35) (Fig. 2a). Kaplan–Meier survival curves demonstrated that the overall survival of the patients with low BASP1 expression was significantly longer than those with high BASP1 expression (Fig. 2b, p = 0.001). These results suggested high BASP1 levels might be an unfavorable factor for patients with cervical cancer.

We further analyzed the correlation between BASP1 levels and the clinicopathological parameters of the patients. As shown in Table 1, we found there was no significant correlation between BASP1 levels and age or pathological differentiation. BASP1 levels correlated significantly with clinical stage (p < 0.001), T classification (larger tumor size, p < 0.001), N classification (lymph node involvement, p < 0.05) and survival or mortality (p < 0.05). These results were confirmed by Spearman’s correlation analysis, as summarized in Additional file 2: Table S3, in which BASP1 levels correlated significantly with clinical stage (p = 0.000), T classification (p = 0.000), N classification (p = 0.023) and survival or mortality (p = 0.002). Thus, the results demonstrated that BASP1 levels correlated with clinical stage, T classification, N classification, and survival or mortality. Our findings suggested BASP1 levels correlated with the clinical aggressiveness of cervical cancer.

**Table 1:** Correlation between BASP1 expression and clinicopathological characteristics of cervical carcinoma

Characteristics	BASP1		Chi square test *p* value	Fisher’s exact test *p* value
	Low no. cases	High no. cases		
Age (years)				
> 45	40	26	0.809	0.862
≤ 45	41	29		
Clinical stage				
Ib1	51	17	0.000	0.000
Ib2	18	11		
IIa	9	14		
IIb	3	8		
III	0	5		
T classification				
T1a1	14	2	0.000	0.000
T1a2	2	0		
T1b1	36	16		
T1b2	17	9		
T2a1	8	3		
T2a2	1	8		
T2b	3	14		
T3a	0	3		
N classification				
N0	62	32	0.023	0.037
N1	19	23		
M classification				
No	81	55	–	–
Yes	0	0		
Pathologic differentiation				
Well	29	19	0.429	0.437
Moderate	26	13		
Poor	26	23		
Survive or mortality				
Survive	68	33	0.002	0.003
Mortality	13	22		

To identify whether BASP1 could serve as a novel prognostic factor, Cox regression analysis was performed, which showed that a high BASP1 level, clinical stage, and pathological differentiation were independent unfavorable prognostic factors (Table 2). Thus, BASP1 abundance correlated significantly with the prognosis of cervical cancer.

**Table 2:** Univariate and multivariate analyses of various prognostic parameters in patients with cervical carcinoma using Cox-regression analysis

	Univariate analysis			Multivariate analysis		
	No. patients	*p*	Regression coefficient (SE)	*p*	Relative risk	95% confidence interval
Clinical stage						
Ib1	68	0.000	0.503 (0.127)	0.000	1.687	1.318–2.160
Ib2	29					
IIa	23					
IIb	11					
III	5					
Pathologic differentiation						
Well	48	0.039	0.431 (0.209)	0.019	1.659	1.088–2.528
Moderate	39					
Poor	49					
Expression of BASP1						
Low expression	81	0.002	1.084 (0.351)	0.037	2.220	1.051–4.692
High expression	55					

### BASP1 regulates tumor growth of cervical cancer

**Fig. 3:**
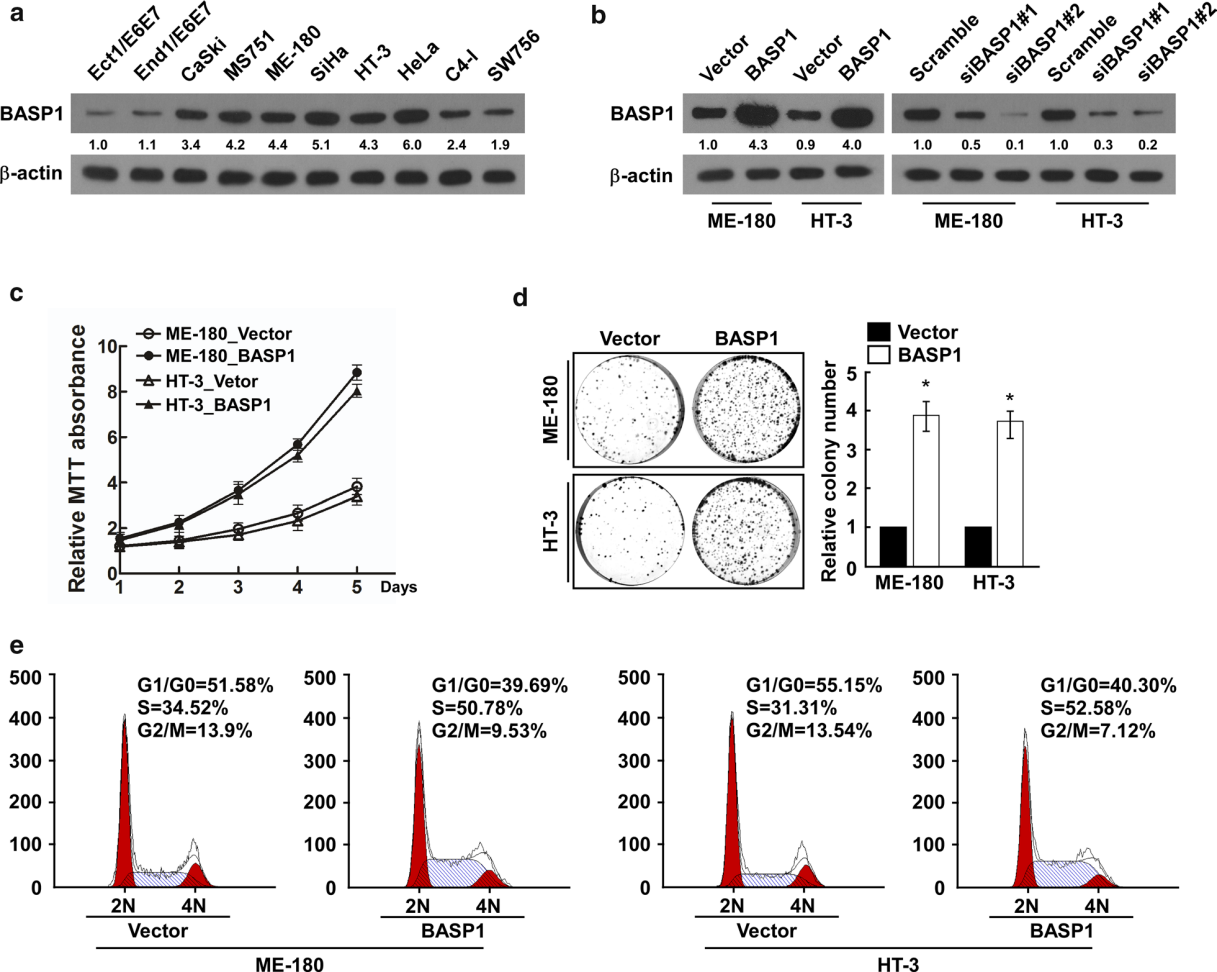
BASP1 was upregulated in cervical cancer cells, and overexpression of BASP1 prompted the proliferation of cervical cancer. **a** Western blot showing BASP1 levels in immortalized ectocervical and endocervical epithelium and cervical cancer cells. **b** Western blot assay confirming the presence of BASP1 in indicated transduced cells. **c** MTT assay showing the effect of BASP1 overexpression on cell proliferation. **d** Colony formation assay showing the effect of BASP1 overexpressed on cell proliferation; representative micrographs (left) and quantification (right) of crystal violet stained cell colonies. **e** Cell cycle analysis showing the effect of BASP1 overexpression on cell proliferation. *p < 0.05, error bars represent the mean ± SD

Brain abundant membrane attached signal protein 1 could serve as a new prognostic factor; therefore, we further determined whether BASP1 regulates tumor growth of cervical cancer. BASP1 levels were determined in cervical cancer cell lines and in two papillomavirus-immortalized normal cervical cell lines. BASP1 was upregulated in the cervical cancer cell lines (Fig. 3a), which was consistent with our previous results for BASP1 abundance in cervical cancer tissues (Fig. 1a, b). We determined the functional role of BASP1 in cervical cancer development by gain or loss of BASP1 in ME-180 and HT-3 cells. Western blotting showed that BASP1 levels were significantly upregulated when cells were transfected with pMSCV-BASP1, and were significantly downregulated when the cells were transfected with a short interfering RNA (siRNA) targeting *BASP1* (siBASP1; Fig. 3b). The MTT assay indicated that overexpression of *BASP1* promoted cervical cancer cell proliferation. A colony formation assay also showed that the cell number increased significantly when *BASP1* was overexpressed (Fig. 3d). These results suggested that BASP1 promotes proliferation of cervical cancer cells. To further confirm that BASP1 regulates cell proliferation, we used cell cycle assays to determine the effect of BASP1 on cell cycle progression, overexpression of *BASP1* increased the proportion of cells in the S phase from 34.52 to 50.78% in ME-180 cells, and from 31.31 to 52.58% in HT-3 cells, with a concomitant decrease in the proportion of cells in the G1/G2/M phases (Fig. 3e).

We confirmed these results by downregulating *BASP1* in the indicated cervical cancer cell lines using siRNAs. The MTT assay showed decreased proliferation of cells transfected with siBASP1 compared to those transfected with a scrambled siRNA (Additional file 3: Figure S1A). The colony formation assay also showed that knockdown of *BASP1* inhibited cellular proliferation (Additional file 3: Figure S1B). We also analyzed the effect of knockdown of *BASP1* on cell cycle progression: The proportion of cells in the S phase decreased from 37.84 to 11.40% in ME-180 cells, and from 35.79 to 12.81% in HT-3 cells, with a concomitant increase in the proportion of cells in the G1/G2/M phases (Additional file 3: Figure S1C).

**Fig. 4:**
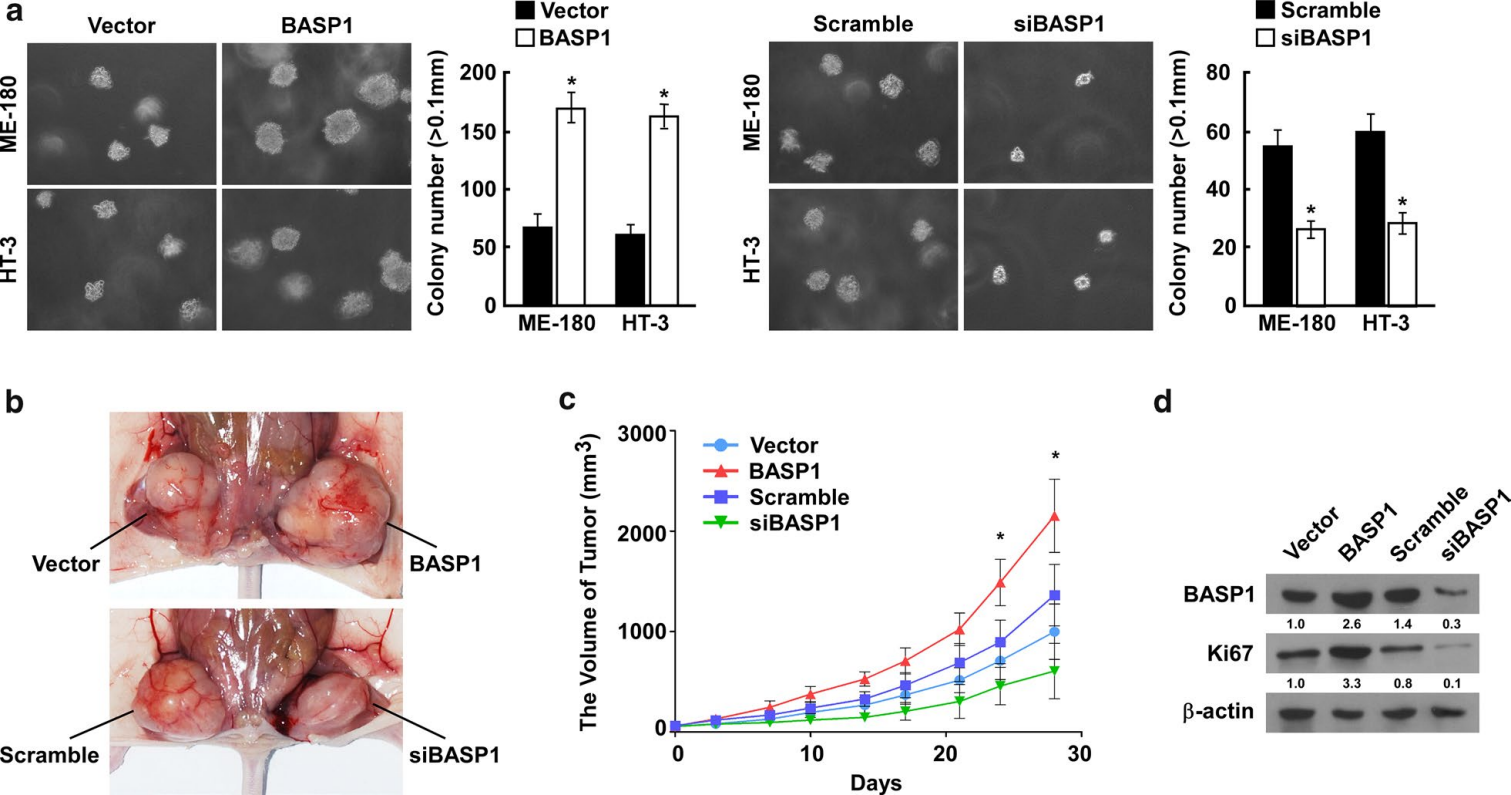
BASP1 promoted tumorigenicity of cervical cancer and tumor growth in vivo. **a** Anchorage-independent growth assay showing the effect of *BASP1* overexpression or knockdown on the tumorigenicity of indicated cervical cells, Representative micrographs (left) and quantification of colonies that were > 0.1 mm (right). **b** The effect of BASP1 on cell growth used xenograft model in nude mice, ME-180 cells with *BASP1* overexpression or knockdown were injected to the subcutaneous sites of nude mice. Representative images of the tumors in nude mice. **c** The effect of BASP1 on cell growth in a xenograft model in nude mice; tumor volumes were measured on the indicated days. **d** Western blot showing Ki67 levels in tumors growth from indicated transduced cells in nude mice. *p < 0.05, error bars represent the mean ± SD

To determine the role of BASP1 in tumorigenicity, we used an anchorage-independent growth assay to determine the effect of BASP1 on tumorigenicity in vitro. *BASP1* overexpression increased the anchorage-independent growth ability of indicated cells significantly, as shown by increased colony numbers and sizes (Fig. 4a). When *BASP1* expression as knocked down, we observed a significant decrease the cells’ anchorage-independent growth ability (Fig. 4a). This suggested that BASP1 promoted the tumorigenicity of cervical cancer.

To confirm that BASP1 regulates cell proliferation and tumorigenicity, we determine the effect of BASP1 on tumor growth in vivo. We transplanted the indicated cells with *BASP1* overexpression or knockdown into a subcutaneous area of nude mice. Overexpression of *BASP1* promoted cervical tumor growth in the nude mice, and downregulation of *BASP1* inhibited it (Fig. 4b, c). Ki67 is a marker for cell proliferation [10]; therefore, we determined Ki67 levels in the tumors grown in the nude mice, and found that Ki67 levels were upregulated in tumors overexpressing *BASP1* and downregulated in *BASP1* knockdown tumors (Fig. 4d). Together, these results suggested that BASP1 promotes tumor growth. This result also supported our clinical investigation, in which BASP1 levels correlated with T classification.

## Discussion

In the present study, we demonstrated that BASP1 plays an important role in cervical cancer. BASP1 was upregulated in cervical cancer, and is a novel unfavorable prognostic factor for patients with cervical cancer. High BASP1 levels correlated with poor clinical outcome. BASP1 also regulates the proliferation and tumorigenicity of cervical cancer; overexpression of *BASP1* promoted cellular proliferation and tumorigenicity and knockdown of *BASP1* had the opposite effect. These results suggested that BASP1 not only serves as a prognostic factor, but also can function as a target for cervical cancer therapy.

We found BASP1 levels were high in cervical cancer, suggesting that *BASP1* may be an oncogene. However, previous reports have shown that BASP1 is downregulated in v-myc-induced transformed cells, and that overexpression of BASP1 inhibits transformation; further analysis showed that BASP1 inhibits the target genes of c-Myc, such as WS5, Q83 and BRAK, suggesting that BASP1 could be a tumor suppressor [11]. Moribe and colleagues used a gene microarray and pyrosequencing to screen genes that are methylated specifically in hepatocellular carcinoma (HCC), and found that *BASP1* is aberrantly methylated in HCC; its expression is low in HCC, and it can function as a useful biomarker for the diagnosis of HCC [12]. MicroRNA miR-191, an onco-miR, is upregulated in transformed human bronchial epithelial cells, and promotes epithelial-mesenchymal transition (EMT) and self-renewal of cancer stem cells of transformed cells. BASP1 is a direct target of miR-191; BASP1 inhibition by miR-191 leads to transactivation of WT1, which activates the Wnt pathway to promote tumor progression [13]. Many genes have been found to play different roles in different kinds of tumors. For example, inhibitor of DNA binding 2 (ID2) is downregulated in breast cancer, in which it inhibits cellular invasion and is a favorable prognostic factor for patients [14]. However, ID2 is upregulated in brain cancer, colon cancer, pancreatic cancer, and prostate cancer, in which it promotes tumor progression, making it an unfavorable prognostic factor [15–18].

We found the opposite role of BASP1 in cervical cancer. Gene set enrichment analyses (GSEA) demonstrated that *BASP1* expression correlated significantly with progression and development of cervical cancer (Additional file 4: Figure S2), revealing that BASP1 is an oncogene in cervical cancer. We further studied whether BASP1 is an unfavorable prognostic factor. Statistical analysis of BASP1 levels in 136 paraffin-embedded cervical cancer tissues suggested a positive correlation between BASP1 levels and clinical stage, T classification, N classification and survival or mortality. Cox regression analysis demonstrated that BASP1 is an independent prognostic factor for patients with cervical cancer, and thus could be used to predict their prognosis.

We also determined the role of BASP1 in the proliferation and tumorigenicity of cervical cancer; overexpression of *BASP1* promoted proliferation, colony formation, cell cycle progression, and tumorigenicity. Knockdown of *BASP1* had the opposite effects. These results suggested that BASP1 regulates the proliferation and tumorigenicity of cervical cancer, making a potential therapeutic target. However, the mechanism by which BASP1 promotes the proliferation and tumorigenicity of cervical cancer requires further study; for example, a chromatin immunoprecipitation assay could identify the target genes of BASP1 associated with cervical cancer.

## Conclusion

We demonstrated that BASP1 is an independent prognostic factor for patients with cervical cancer that promotes cervical cancer growth.

## Methods and materials

### Cell culture and transfection

Human cervical cancer cell lines Ca Ski, MS751, ME-180, SiHa, HT-3, HeLa, C4-I and SW756 were cultured in Dulbecco’s modified Eagle’s medium (DMEM) (Natocor) supplemented with 10% fetal bovine serum (FBS) (GIBCO). Ectocervical Ect1/E6E7 and endocervical End1/E6E7 cells were cultured in Keratinocyte-Serum Free medium (GIBCO-BRL, 17005-042, USA) supplemented with 0.1 ng/mL human recombinant EGF, 0.05 mg/mL bovine pituitary extract (BPE, Sigma) and 0.4 mM CaCl_2_ (Natocor). The cells were maintained in 5% CO_2_ at 37 °C.

The full-length BASP1 cDNA was cloned into vector pMSCV-puro; pMSCV-empty vector was used as the negative control. pMSCV-BASP1 and pMSCV-empty vector were cotransfected with the IK packaging plasmid into 293T cells using the calcium phosphate transfection method. Supernatants were collected at 48 h after transfection, and infected with cervical cells for 12 h in the presence of polybrene (2.5 μg/mL). Puromycin was used to select the transfected cell lines.

For BASP1 knockdown experiments, two siRNAs for *BASP1* and one scrambled siRNA were synthesized by Guangzhou RiboBio Co (Guangzhou, China). The sequences used to downregulate *BASP1* were: siBASP1#1: 5′CGGGATCCATGGGA3′, siBASP1#2: 5′CGGAATTCTCACTCT3′. 50 nM of the siRNA was transfected into ME-180 and HT-3 cells using the Lipofectamine RNAiMAX Transfection Reagent (Life Technologies).

### Patients and tissue samples

Six cervical cancer tissues and matched adjacent normal cervical epithelium tissues were obtained from the First Affiliated Hospital of Sun Yat-sen University. Guangzhou. These samples were snap-frozen immediately and stored in liquid nitrogen until use. To further analyze the relationship between BASP1 expression and the clinicopathological parameters, a cohort of 136 paraffin-embedded cervical cancer tissues was used. These tissues were diagnosed histopathologically and clinically at the First Affiliated Hospital of Sun Yat-sen University. For the use of above clinical samples for research purposes, prior patient’s consent and approval from the Institute Research Ethics Committee of the First Affiliated Hospital of Sun Yat-sen University were obtained. The detailed clinicopathological parameters are shown in Additional file 5: Table S1.

### Western blotting and immunohistochemistry (IHC)

Western blotting was performed according to standard methods, as described previously [19], using anti-BASP1 (ab101855, Abcam), anti-Ki67 (sc-7846, Santa Cruz) antibodies. The membranes were stripped and reprobed with anti-β-actin antibodies as a loading control. The band intensity was analyzed using Image J software.

IHC was performed as described previously [20] using the anti-BASP1 antibody (ab101855, Abcam). The results of staining were scored independently by two pathologists blinded to the clinical outcome, and were based on both the proportion of positively stained tumors cells and the intensity of staining. The proportion of stained tumor cells was scored as follows: score 0, no positive cells; score 1, up to 25% positive cells; score 2, 26–50% positive cells; score 3, 51–75% positive cells; score 4, over 75% positive cells. The intensity of staining was determined as: 0 (no staining), 1 (light yellow = weak staining), 2 (yellow brown = moderate staining), and 3 (brown = strong staining). The staining index (SI) was determined as the product of staining intensity × percentage of positive tumor cells. Cutoff values for high and low expression of BASP1 were chosen based on a measurement of heterogeneity using the log-rank test with respect to overall survival. An SI score of greater than or equal to 6 was considered to be high expression, and an SI score of less than 6 was considered low expression.

### Cell proliferation and cell cycle assay

To examine the role of BASP1 in the proliferation of cervical cancer cells, an MTT assay and colony formation assay were performed using previous described methods [21].

Cell cycle analysis was performed using a previously described method [21]. Briefly, cells were harvested and washed in cold PBS followed by fixation in 70% alcohol overnight at 4 °C. After washing with cold PBS three times, the cells were resuspended in PBS solution with 20 μg/mL propidium iodide (Sigma) and μg/mL RNase A for 30 min at 37 °C. Samples were then analyzed using a FACSCalibur cytometer (Becton–Dickinson).

### Anchorage-independent growth assay

An anchorage-independent growth assay was performed according to a previously described method [21, 22]. Briefly, 500 cells were suspended in 2 mL of complete medium containing 0.3% agar (Sigma). The agar–cell mixture was plated on top of a solid bottom layer containing 1% complete medium agar mixture. After 10 days, colonies that contained more than 50 cells or were larger than 0.1 mm in diameter were counted. The experiment was performed in triplicate.

### Growth of tumor xenografts in nude mice

Animal studies were performed according to institutional guidelines. BALB/c nude mice (4–5 weeks old) were used to make a xenograft model using ME-180 cervical cancer cell lines. Five mice were assigned randomly to each group and injected with ME-180 transfected with pMSCV-BASP1, pMSCV-empty, scramble siRNA, or BASP1 siRNA, and used to determine the role of BASP1 in tumor progression. 1 × 10^6^ cells were injected into the subcutaneous sites of nude mice. The tumor volume was calculated every 2 days for 1 month. The tumors were excised and subjected to protein extraction to determine Ki67 levels using western blotting.

### Statistical analysis

All statistical analyses were performed using the SPSS 19.0 statistical software package. Results are presented as the mean ± standard deviation (SD) for at last three repeated individual experiments for each group. Chi square and Fisher’s exact tests were used to analyze the relationship between BASP1 levels and clinicopathological parameters. Bivariate correlations between variables were calculated using Spearman’s rank correlation coefficients. The survival curve was plotted using Kaplan–Meier survival analysis and compared using a log-rank test. Univariate and multivariate Cox regression analyses were used to estimate the significance of various variables for survival. A value of p < 0.05 was considered significant in all cases.

## Additional files

**Additional file 1: Table S2.** The expression of BASP1 in cervical carcinoma.

**Additional file 2: Table S3.** Spearman correlation analysis between BASP1 and clinicopathological factors.

**Additional file 3: Figure S1.** Knockdown of BASP1 inhibited the proliferation of cervical cancer. (A) MTT assay showing the effect of BASP1 knockdown on cell proliferation. (B) Colony formation assay showing the effect of BASP1 knockdown on cell proliferation, Representative micrographs *(left)* and quantification *(right)* of crystal violet stained cell colonies. (C) Cell cycle analysis indicating the effect of BASP1 knockdown on cell proliferation. *p < 0.05, error bars represent mean ± SD.

**Additional file 4: Figure S2.** Gene set enrichment analyses (GSEA) demonstrating that BASP1 expression correlates significantly with progression and development of cervical cancer. ES: enrichment score, NES: normalized enrichment score.

**Additional file 5: Table S1.** Clinicopathological characteristics of cervical carcinoma patient samples.
