# Supplementary material for: High brain acid soluble protein 1(BASP1) is a poor prognostic factor for cervical cancer and promotes tumor growth
Source: Cancer Cell Int. 2017 Oct 24;17:97. doi: 10.1186/s12935-017-0452-4 (PMC5655910; doi:10.1186/s12935-017-0452-4)
Supplement: Supplementary file 1 — Additional file 1: Table S2. The expression of BASP1 in cervical carcinoma. [file 12935_2017_452_MOESM1_ESM.docx]

**Supplemental Table 2 The expression of BASP1 in cervical carcinoma**

| **Expression of BASP1** |  |
| --- | --- |
| Negative | 2 (1.5%) |
| Positive | 134 (98.5%) |
| Low expression | 81 (59.6%) |
| High expression | 55 (40.4%) |
