# Supplementary material for: High brain acid soluble protein 1(BASP1) is a poor prognostic factor for cervical cancer and promotes tumor growth
Source: Cancer Cell Int. 2017 Oct 24;17:97. doi: 10.1186/s12935-017-0452-4 (PMC5655910; doi:10.1186/s12935-017-0452-4)
Supplement: Supplementary file 2 — Additional file 2: Table S3. Spearman correlation analysis between BASP1 and clinicopathological factors. [file 12935_2017_452_MOESM2_ESM.docx]

**Supplemental Table 3 Spearman correlation analysis between BASP1 and clinical pathologic factors**

| **Variables** | **BASP1 Expression Level** | |
| --- | --- | --- |
|  | **Spearman Correlation** | ***P*-Value** |
| **Clinical stage** | 0.385 | 0.000 |
| **T classification** | 0.423 | 0.000 |
| **N classification** | 0.195 | 0.023 |
| **Survive or Mortality** | 0.269 | 0.002 |
