# Supplementary material for: High brain acid soluble protein 1(BASP1) is a poor prognostic factor for cervical cancer and promotes tumor growth
Source: Cancer Cell Int. 2017 Oct 24;17:97. doi: 10.1186/s12935-017-0452-4 (PMC5655910; doi:10.1186/s12935-017-0452-4)
Supplement: Supplementary file 5 — Additional file 5: Table S1. Clinicopathological characteristics of cervical carcinoma patient samples. [file 12935_2017_452_MOESM5_ESM.docx]

**Supplemental Table 1 Clinicopathological characteristics of cervical carcinoma patient samples**

|  | **Number of cases** |
| --- | --- |
| **Age(years)** |  |
| > 45 | 66 |
| < 45 | 70 |
| **Clinical Stage** |  |
| Ib1 | 68 |
| Ib2 | 29 |
| IIa | 23 |
| IIb | 11 |
| III | 5 |
| **T classification** |  |
| T1a1 | 16 |
| T1a2 | 2 |
| T1b1 | 52 |
| T1b2 | 26 |
| T2a1 | 11 |
| T2a2 | 9 |
| T2b | 17 |
| T3a | 3 |
| **N classification** |  |
| N0 | 94 |
| N1 | 42 |
| **M classification** |  |
| Yes | 0 |
| No | 136 |
| **Pathologic Differentiation** |  |
| Well | 48 |
| Moderate | 39 |
| Poor | 49 |
| **Survive or Mortality** |  |
| Survive | 101 |
| Mortality | 35 |
